# Heterozygous genome assembly via binary classification of homologous sequence

**DOI:** 10.1186/1471-2105-16-S7-S5

**Published:** 2015-04-23

**Authors:** Paul M Bodily, M Stanley Fujimoto, Cameron Ortega, Nozomu Okuda, Jared C Price, Mark J Clement, Quinn Snell

**Affiliations:** 1 Computer Science Department, Brigham Young University, 3361 TMCB PO Box 26576, Provo, UT, 84602-6576, USA

**Keywords:** diploid genome assembly, machine learning, scaffolding

## Abstract

**Background:**

Genome assemblers to date have predominantly targeted haploid reference reconstruction from homozygous data. When applied to diploid genome assembly, these assemblers perform poorly, owing to the violation of assumptions during both the contigging and scaffolding phases. Effective tools to overcome these problems are in growing demand. Increasing parameter stringency during contigging is an effective solution to obtaining haplotype-specific contigs; however, effective algorithms for scaffolding such contigs are lacking.

**Methods:**

We present a stand-alone scaffolding algorithm, ScaffoldScaffolder, designed specifically for scaffolding diploid genomes. The algorithm identifies homologous sequences as found in "bubble" structures in scaffold graphs. Machine learning classification is used to then classify sequences in partial bubbles as homologous or non-homologous sequences prior to reconstructing haplotype-specific scaffolds. We define four new metrics for assessing diploid scaffolding accuracy: contig sequencing depth, contig homogeneity, phase group homogeneity, and heterogeneity between phase groups.

**Results:**

We demonstrate the viability of using bubbles to identify heterozygous homologous contigs, which we term homolotigs. We show that machine learning classification trained on these homolotig pairs can be used effectively for identifying homologous sequences elsewhere in the data with high precision (assuming error-free reads).

**Conclusion:**

More work is required to comparatively analyze this approach on real data with various parameters and classifiers against other diploid genome assembly methods. However, the initial results of ScaffoldScaffolder supply validity to the idea of employing machine learning in the difficult task of diploid genome assembly. Software is available at http://bioresearch.byu.edu/scaffoldscaffolder.

## Background

Efficient and accurate genome assemblies facilitate effective data-driven solutions in fields such as personalized medicine, genetic engineering, and even next-generation digital information storage [1]. A genome contains all of the genetic information needed for an organism to live and represents a trove of data for seeking to understand the complex mechanisms governing all life. Proper analysis of these data presupposes a correctness of the reconstructed genomic sequence, which continues to motivate the need for assembly algorithms which produce assemblies from next-generation sequence data with greater completeness and correctness.

Genome assemblers have traditionally been designed to assemble haploid genomes [2-4]. This was motivated in the first place by the vast array of monoploid bacterial genomes being sequenced and then later on by the ease with which the two haplotypes of many diploid species could be made homogenous or *homozygous *enough (via inbreeding) to nearly approximate a monoploid specimen. Thus initial *de novo *assembly algorithms were designed to essentially ignore any variation that may have existed between haplotypes.

Several attempts have been made to assemble highly-polymorphic genomes [5-9]. Obtaining a homozygous diploid specimen can in some cases make the specimen inviable, not to mention the time, resources, and ethical concerns that are also often inherent in the inbreeding process. In all of these cases, traditional assembly techniques are poorly equipped to handle the challenges posed by heterozygosity [10]. Of increasing importance are the questions targeting minute genetic variations that are ultimately responsible for a phenotype or disease in populations as well as individuals. These emphases issue a renewed challenge to develop algorithms to produce high-resolution diploid genomes, including *phased *haplotypic variation (*phasing *is the process of ensuring that variants on the same haplotype are assembled together).

There exist three primary classes of approaches to this problem. The first approach is to solve *haplotype phasing problem*, which takes (unphased) haplotype data (e.g., SNP chip data) and generates phased haplotypes using parsimony or maximum-likelihood estimation methods [11-13]. In doing so, population data may be used to estimate haplotype frequencies, as well as to impute ungenotyped loci [14].

A second common approach is to address the challenge in terms of the *haplotype assembly problem *[15] or simply the *individual haplotyping problem *[16], which takes reads as input in order to first call variants and then phase them. Variants are generally called through *mapping *or aligning reads (based on sequence similarity) to a previously assembled reference sequence for the individual's species [17-20]. Most methods generally do not involve assembly (see [21] for an exception), but rather determine small-scale variation based on loci where the aligned reads suggest a different nucleotide than that present in the reference [22,23]. The primary advantage of the haplotype assembly approach is its use of a reference to ensure access to all regions of a complete, highly manicured reference. However, results from even just the mapping phase vary widely both by algorithm and based on parameter settings [24]. Mappability is also affected by sequencing errors and heterozygosity [25]. Mapping to a reference also fails to capture large-scale rearrangements.

Because of the problems inherent in mapping and in using a reference, the problem of true *de novo *diploid genome assembly, meaning the complete assembly of two haplotypes from sequenced reads without the use of a reference, has begun recently to see increased emphasis (e.g., see Discovar [26] and Hapsembler [27]). We present ScaffoldScaffolder, a diploid genome assembly approach which includes a newly developed scaffolding module to resolve haplotype-specific scaffolds.

### Genome assembly background

All DNA sequencing technologies to date have imposed constraints on the length of fragments that can be sequenced. This then requires the genome to be broken into small pieces and then algorithmically reassembled again from the sequenced fragments. There are two broad families of assembly or *contigging *algorithms: those which employ an Overlap Layout Consensus (OLC) and those which employ a de Bruijn Graph [4]. OLC algorithms, such as Newbler (454 Life Sciences), attempt to reconstruct larger sequences (termed *contigs*) by maximally overlapping reads. This approach generally produces more complete, accurate assemblies, but is *O*(*n*^2^), meaning the runtime increases exponentially with the size of the input data. De Bruijn Graph assemblers, such as SOAPdenovo [28], are *O*(*n*), meaning their run-time increases linearly with the size of the input data, however, the results are often more fragmented than OLC assemblers. This speed-accuracy tradeoff is a non-trivial decision in each *de novo *assembly.

OLC assemblers have historically been favored as well for their flexibility with handling errors or *heterozygosity *(i.e., variation between haplotypes). Whereas de Bruijn Graph assemblers must sacrifice a great deal in efficiency in order to consider mismatches or insertion/deletions (indels) in an overlapped read, OLC assemblers can be parameterized to efficiently align mismatched regions. Errors and heterozygosity can thus be easily ignored by simply accepting the most *common *nucleotide at a given locus from those present in the pileup (hence *consensus*).

**Figure 1:**
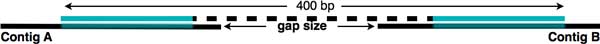
**Paired Read**. Shown is a 400-bp insert whose ends have been sequenced. When aligned to the contig set, the sequenced ends aligned at specific locations in Contigs *A *and *B*, allowing inference about the relative orientation of and distance between the two contigs.

Neither assembly approach is natively designed to be able to resolve repetitive sequence or to overcome deficiencies in data sampling. Thus, regardless of the method used, the contigging algorithm produces a set of contigs that is considerably larger than the haploid number that would be ideally recovered. To reduce the set of sequences further, contigs can be positioned and oriented relative to one another using long "anchoring" fragments. These *paired-read *fragments, whose length is approximately known, are too long to be sequenced end to end, but short snippets can be sequenced from either end. Inasmuch as these end sequences find matches in the contig set, the paired-read fragment can "anchor" two contigs at an approximate distance and in a specific relative orientation (see Figure 1). This process is termed *scaffolding *or *meta-contigging*.

The set of contigs and their scaffoldings can be modeled as a graph from which must be elucidated the subset of non-conflicting, highly-supported scaffoldings that represent the correct genomic reconstruction. The graph is made complex by an array of confounding effects including: inaccurate contig assembly; erroneous scaffolding evidence deriving from error-prone read sequencing; the collapsing of highly-repetitive DNA elements into single contigs; possible sample contamination; insufficient data sampling; and insufficient paired read evidence.

## Methods

Our method involves two steps: 1. assembling reads so as to preserve haplotype-specificity in the assembled contig set and 2. scaffold contigs into linear haploid scaffolds using a modified scaffolding algorithm.

### Assembly

In cases where the haplotypic variation is significant, heterozygosity can be preserved by requiring very stringent overlaps in the OLC process. As overlap stringency parameters are often relaxed in order to merge haplotypes, requiring near perfect overlaps will disallow the merging of haplotypes in favor of more accurate, complete diploid contig assembly. This is essentially equivalent to assuming that haplotypes represent different--though similar--molecules (which they do), and should therefore assemble separately. In theory this optimization lends itself nicely to using de Bruijn graph assemblers which perform optimally when perfect overlaps are assumed.

Imposing more strict overlap requirements during assembly produces a contig set which contains a more biologically accurate representation of the genome: a set of contigs which represent sequence that is homozygous between haplotypes (*homotigs*) and a set of contigs which represent sequence unique to one haplotype in regions where the genome is heterozygous (*heterotigs*). Some of the homotigs may represent repetitive sequence. Nonetheless, by ensuring coverage that is sufficiently high, we may assume that by this approach, every sequence in the genome (whether it belongs to a homozygous or heterozygous region) is assembled in tact in our set of contigs.

### Scaffolding

We need to state a few more assumptions about the dataset at this point. First, to simplify the problem, we will assume that there are no large-scale rearrangements between haplotypes. Although such rearrangements happen and several can be algorithmically identified [29], our proposed solution assumes that variation between haplotypes is small-scale. Essentially this allows us to assume that heterozygous sequence that is sequentially similar will be reconstructed in the same orientation at the same position on opposite haplotypes.

This leads to our second assumption, which is that for each heterotig *a *in the contig set (deriving from haplotype *A*), there exists exactly one homologous heterotig *b*, representing the reconstructed sequence on haplotype *B *opposite *a*. We will say that two such heterotigs *a *and *b *represent a *homolotig pair*.

**Figure 2:**
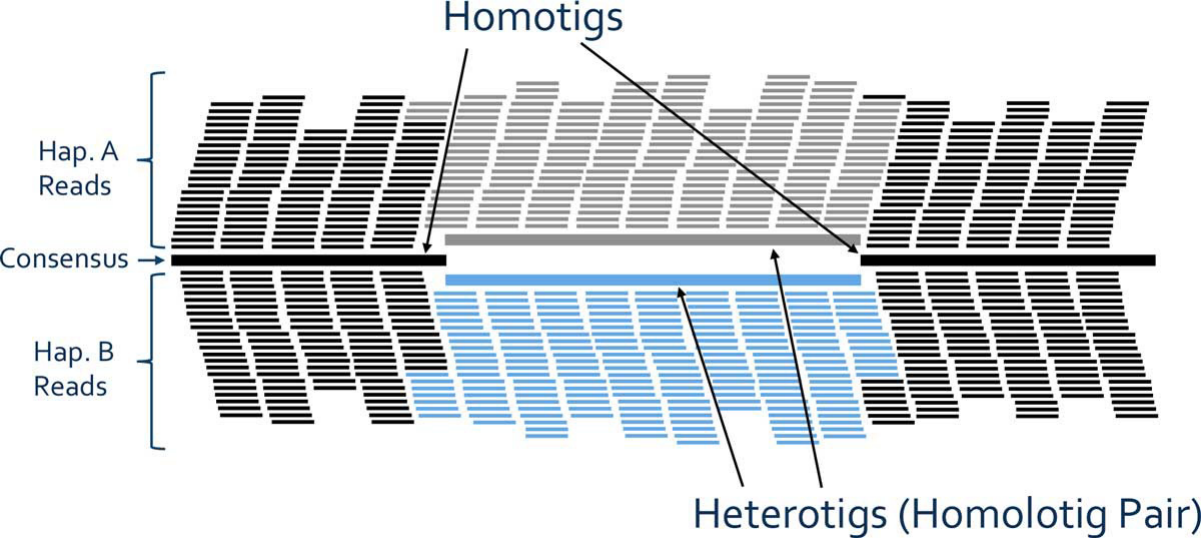
**A Heterozygous Bubble**. *Homotigs *are formed from homozygous sequence, where read pileups from both haplotypes have the same consensus sequence. *Heterotigs *are formed from heterozygous sequences, where read pileups from each haplotype have a unique consensus due to variation. Inasmuch as two heterotigs are homologous, we say that they form a *homolotig pair*.

Third, we will assume that each homolotig pair exists in the larger context of a heterozygous "bubble" [30,31], meaning that both heterotigs in the pair are flanked by a common homotig on their 5' end and by a different common homotig on their 3' end (see Figure 2). This assumption is based on the general practice of assembly algorithms to discontinue contig elongation when the read pileup is suggestive of two possible reconstructive paths, as would happen at the junction between homozygous and heterozygous sequence. We assume that as long as contiguous sequence is homologous it will be constructed into a single homotig. Where there is heterozygosity, there will be a bifurcation into two homologous heterotigs. Where there is no longer variation between haplotypes, the reconstruction of the two haplotypes will re-merge as a single homotig, thus completing the bubble.

The diploid heterozygous genome assembly problem thus takes on the form of elucidating from the noisy, bidirected scaffold graph an interleaving pattern of single homotigs and homolotig pairs. It should be noted that contigs representing repetitive DNA elements pose a particularly difficult challenge and where such contigs are identified (both by contributive read coverage and by abnormally high total vertex degree in the scaffold graph), we do not attempt to include them in the reconstructed genome.

### ScaffoldScaffolder

**Figure 3:**
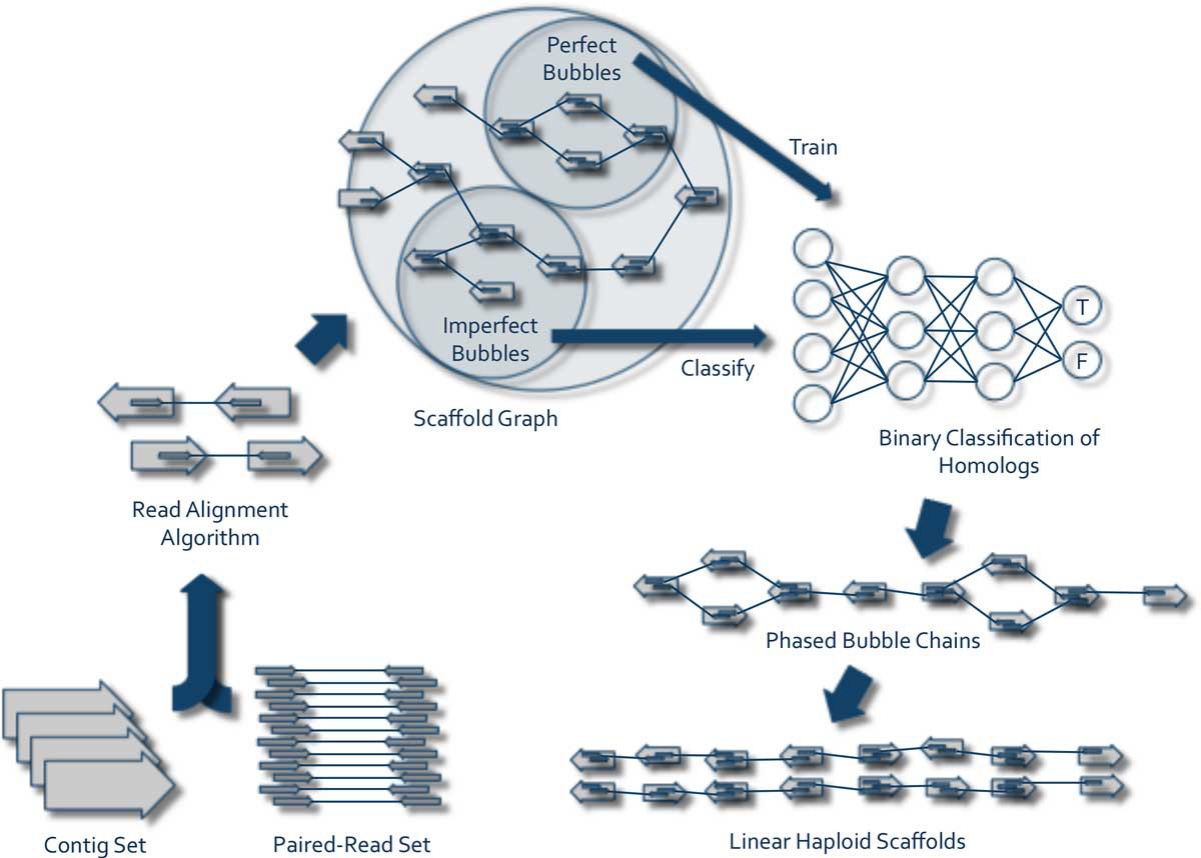
**ScaffoldScaffolder Overview**.

ScaffoldScaffolder was originally developed as a greedy, stand-alone scaffolding algorithm [32]. In expanding its functionality, we have introduced a module capable of scaffolding diploid heterozygous genomes which have been assembled according to the above-mentioned criteria. The algorithm (implemented in Java) consists of 6 principal steps (see Figure 3):

1. *Input*. The algorithm takes as input a contig set in FASTA format and a set of paired read files in FASTA, FASTQ, or several other formats. Alternatively, ScaffoldScaffolder can operate given a FASTA contig file and a set of alignment output files in SAM, Bowtie, or BLAST output formats. If this is the case, the algorithm will skip the Read Alignment step.

2. *Read alignment*. Paired reads are aligned to the contig set using any read alignment algorithm which outputs in one of the following formats: SAM, Bowtie, or Blast (default aligner is Bowtie).

3. *Scaffold graph construction*. A scaffold graph, as described in the Background section is created and edges are created and weighted from evidence in the paired read alignment output.

4. *Binary classification of homologs*. A binary classifier is trained to identify homolotig pairs as found in "perfect" or easily recognizable bubbles. This classifier is then used to identify additional homolotig pairs in "imperfect" bubbles.

5. *Bubble chain elucidation*. Remaining scaffoldings are considered greedily for linking bubbles together into chains. Paired reads from larger insert libraries are used to phase haplotypes and further conjoin scaffolds.

6. *Linear haploid scaffold formation*. The algorithm outputs two FASTA files, each containing one of two linear, phased haplotypes from each scaffold. Several other metadata files are optionally printed to provide information used in the scaffolding process.

We will consider each of these steps in more detail.

#### Input

The minimum input required to run ScaffoldScaffolder is a FASTA file containing stringently assembled contigs and a "reads info file," which is a file formatted identical to the SOAPdenovo configuration file and contains information for each paired read library including the file locations, the average insert size, and an order in which the library is to be used for scaffolding. The paired read files themselves can be in nearly any format because for the read alignment step, ScaffoldScaffolder uses a user-configured third-party read aligner to align the reads. All that matters is that following the read alignment step, ScaffoldScaffolder will look for output alignment files in one of three formats: SAM, Bowtie or Blast. Alternatively, the user can indicate the alignment files already exist and ScaffoldScaffolder will not attempt to reperform the alignment.

#### Read alignment

When aligning the paired reads to the contigs, ScaffoldScaffolder allows the user to specify which alignment algorithm should be used and with which parameters. A "mapper default" configuration file is provided which can be easily edited to create several custom alignment configurations for one or more alignment algorithms. Each configuration is given a user-specified identifier that can then be easily selected at runtime. The default configuration file includes configurations for Bowtie, Bowtie2, GSnap, and BLASTn (one of which must be separately installed prior to use by ScaffoldScaffolder), however any preferred mapper can be configured, provided that it outputs in one of the accepted formats. A flag allows the user to instruct ScaffoldScaffolder to use "existent mappings," which not only allows the scaffolder to run on alignment files, but has the added benefit of allowing users to run ScaffoldScaffolder repeatedly with different parameterizations without ever having to rerun the read alignment step. ScaffoldScaffolder is not enabled to run on multiple processors. However, a 'procs' parameter can be set to allow third-party algorithms that are so enabled to run on the input number of threads.

#### Scaffold graph construction

On conclusion of the read alignment phase, ScaffoldScaffolder initializes an empty graph containing a vertex for each contig. Then for each paired read for which both ends had a reported alignment, a new weighted edge is created or if the corresponding edge already exists, its weight is incremented (each edge is bidirected in that it defines an orientation for both contigs). The final scaffolding distance is the computed average of the distances suggested by the alignment of each supporting paired read. ScaffoldScaffolder allows for ends to be multiply mapped, but it is suggested that multiply-mapped reads be suppressed by the alignment algorithm in order to more effectively manage memory. In creating the scaffold graph, an optional flag allows the user to instruct ScaffoldScaffolder to output a metafile containing the status of how each paired read was used in the scaffold graph. Each paired read status includes information about if and where each end mapped, whether the pair was used to link two contigs, and whether both ends mapped within a contig (and if so, the distance between the ends). This file is formatted similar to the PairStatus file of the 454 Newbler assembler.

#### Binary classification of homologs

Once the graph is constructed, ScaffoldScaffolder seeks to algorithmically select a subset of contig scaffoldings according to one of two algorithms. The first is a simple greedy algorithm which includes edges in the final subset provided they do not conflict with previously included edges. This algorithm proceeds iteratively in that only a single rank of libraries is considered at each iteration of the greedy algorithm. It is also important to note that at each iteration, a contig may be scaffolded only once in the 5' direction and once in the 3' direction. Thus, a conflicting edge from a contig is any edge that would scaffold that contig in a direction in which it has already been scaffolded. For this reason, ScaffoldScaffolder works optimally when at least one of the libraries has small insert size (500 bp or smaller) in order to resolve more local contig scaffoldings prior to attempting larger-scale scaffoldings. The greedy algorithm makes no attempt to reconcile homologous sequence, but rather greedily chooses reconstructions where multiple possibilities exist. For this reason, the greedy algorithm is not designed to be used on heterozygous diploid datasets.

ScaffoldScaffolder is equipped with a second scaffold graph reduction algorithm, called the "bubble finder solution" (BFS), which is designed specifically to scaffold diploid contig sets. The BFS begins by algorithmically searching the scaffold graph for bubbles. In the context of the scaffold graph, we will differentiate between a "perfect" and an "imperfect" bubble. A perfect bubble is defined as two contigs *a *and *b *(presumably a homolotig pair), each of which have a single candidate scaffolding (i.e. no conflicting edges) to a common contig in both the 5' and 3' directions (see Figure 3). That is, contig *a *has a single scaffolding (*a, c*_1_) in the 5' direction of *a *and a single scaffolding (*a, c*_2_) in the 3' direction; contig *b *has a single scaffolding (*b, c*_1_) in either the 5' or 3' direction of *b *and a single scaffolding (*b, c*_2_) in the opposite direction. Contigs *c*_1 _and *c*_2 _(presumably homotigs) must have identical orientations in each of the scaffoldings in which they appear and are additionally required by our definition of a perfect bubble to have no conflicting scaffoldings with these scaffoldings. Note that two perfect bubbles can share a homotig, but cannot otherwise overlap. A perfect bubble is therefore a construct in our scaffold graph model which in all probability represents a true heterozygous bubble, referring again to the sequence of homozygous, heterozygous, homozygous regions. (For both the greedy and BFS algorithms, a minimum support may also be specified for scaffold graph edges, below which an edge will essentially be considered nonexistent. The default is 2.)

There are several characteristic features which we expect to be consistent for all true bubbles, particularly features of the pair of homozygous heterotigs. These include the ratio of the heterotig lengths, the ratio of heterotig sequence depths, some normalized measurement of their overall sequence similarity, and the ratio of each sequence depth to the average sequence depth for all contigs [30]. Table 1 gives more precise definitions to each of these features as well as their expected values both for a valid homolotig pair and for a random pair of non-homologous contigs. Note that in order to calculate sequence depth ratios, the user must provide ScaffoldScaffolder with a contig depth file detailing the sequence depth for each contig. In the absence of this information, the algorithm will still proceed, but the power of the classifier will be limited. Sequence similarity for two contigs is computed as a function of the BLASTn results from aligning the two sequences and thus BLASTn must be installed as a prerequisite to using this module. This then represents a feature vector on which we can attempt to classify true versus false homolotig pairs.

**Table 1:** Feature values for classification of homologous contig pairs

Feature Description	Definition	Expected Value for Homologous Pair	Expected Range for Non-Homologous Pair
Length Ratio	$\frac{min\left(seqALen,\;seqBLen\right)}{max\left(seqALen,\;seqBLen\right)}$	≈1	0 < × ≤ 1
Depth Ratio	$\frac{min\left(seqADep,\;seqBDep\right)}{max\left(seqADep,\;seqBDep\right)}$	≈1	0 < × ≤ 1
% Identical Matches	*pidentFromBLASTnAlignment*	≈100	0 ≤ × ≪ 100
% Length Alignment	$\frac{lengthFromBLASTnAlignment}{min\left(seqALen,\;seqBLen\right)}$	≈100	0 ≤ × ≪ 100
Seq A Depth Proportion to Mode	$\frac{seqADep}{ModeOfAllSequencesDepths}$	$\approx \frac{AverageHaploidSequenceDepth}{ModeOfAllSequencesDepths}$	0 < x
Seq B Depth Proportion to Mode	$\frac{seqBDep}{ModeOfAllSequencesDepths}$	$\approx \frac{AverageHaploidSequenceDepth}{ModeOfAllSequencesDepths}$	0 < x

We assume therefore that perfect bubbles accurately predict homolotig pairs. ScaffoldScaffolder then trains a binary classifier on homolotig pairs as found in perfect bubbles using our defined feature vector. The algorithm implements the Weka machine learning framework, allowing the user to specify at runtime any one of the classifiers in the weka.classifiers.functions package, including both a backpropagation multilayer perceptron classifier and a number of support vector machine implementations.

#### Bubble chain elucidation

There are presumably many partial or "imperfect" bubbles in the scaffold graph, which are either missing edges or which have conflicting edges as a result of insufficient data, erroneous alignments, or other biological ambiguities. We can then consider each of these imperfect bubbles in decreasing order of "perfectness" and use our classifier to predict whether or not these latter bubbles represent true heterozygous bubbles as indicated by whether or not the purported homolotig pair is classified as valid. An imperfect bubble, any of whose constituent edges either conflicts with already included edges or *is *an already included edge, is disallowed from being classified a true bubble. This guarantees that the resulting subgraph consists entirely of bubbles and/or chains of bubbles (i.e., bubbles which share common homotigs).

Remaining scaffoldings are then considered greedily (as defined by the simple greedy algorithm above) for linking bubbles and remaining contigs together into chains. As with the greedy algorithm, this algorithm proceeds iteratively, with bubbles being formed and predicted in the first iteration. All iterations past the first use paired reads to link existing bubble chains and scaffolds into larger chains. At each iteration, paired reads mapping within chains are used to phase homolotig pairs so that the final product is a set of phased bubble-chain scaffolds.

#### Linear haploid scaffold formation

From phased bubble chains, ScaffoldScaffolder outputs two FASTA files, each containing one of two linear, phased haplotypes for each scaffold. A linear, phased haplotype is constructed by considering homotigs together with heterotigs from one of the phased haplotypes. ScaffoldScaffolder can optionally output the bubble chain graph in DOT format (viewable with Graphviz) and an additional parameter will additionally display excluded edges as dashed edges.

### Quantitative analysis

We developed four methods for internally assessing the performance of our homolotig classifier and subsequent phasing algorithm. In defining these methods, we use the following definitions. Let *x *represent an arbitrary read. Let *c *simultaneously represent an arbitrary contig as well as the set of reads {*x* | × ∈ *c*} that constitute *c*. For synthetic haplotypes A and B, let *A *and *B *be the set of reads belonging to synthetic haplotypes A and B respectively (i.e., *A *= {*x* | × ∈ *A*} and *B *= {*x* | × ∈ *B*}). Let *L*(*A*) represent the length of synthetic genome A (note that in our analyses, *L*(*A*) = *L*(*B*)). Let *g *represent an arbitrary set of contigs that are phased together (i.e., *g *= {*c | c *∈ *g*}). All analyses were performed for contigs of length greater than 100 bases.

### Contig sequencing depth

The contig sequencing depth refers to the average number of reads that contribute to the consensus at each position in the contig. Reads contributing to homotigs derive from two haplotypes and should therefore reflect a diploid sequencing depth. Reads contributing to heterotigs derive from only one of the two haplotypes and should therefore reflect a haploid sequencing depth. To the extent that predicted heterotigs have a greater-than-haploid sequencing depth, we would suspect a failure of haplotype segregation during assembly. We plot the density of *sequencing depth *values for three classes of contigs: heterotigs used in training, for contigs classified as heterotigs, and for contigs classified as homotigs.

### Contig homogeneity

Properly assembled heterotigs should also exhibit strong homogeneity in the source of their constituents. Insofar as haplotypes segregate properly during assembly, resulting heterotigs should be composed entirely of reads from one haplotype or entirely of reads from the complementary haplotype. For each contig class, we plot a density of *contig homogeneity *values. We define contig homogeneity for a contig *c *as the ratio of reads deriving from haplotype A, calculated as

$$hom_{conting}\left(c\right)=\frac{\left|\left\{x|x\in c\wedge x\in A\right\}\right|}{\left|\left\{x|x\in c\right\}\right|}$$

For contigs trained or classified as heterotigs, we would expect to see peaks near 0 and 1. Homotigs should peak near 0.5.

### Phase group homogeneity

To assess the accuracy of phasing, we analyze the ratio of haplotype A reads in each group of phased contigs, *g*:

$$hom_{phase}\left(g\right)=\frac{\sum_{c\in g}|\left\{x|x\in c\wedge x\in A\right\}|}{\sum_{c\in g}|\left\{x|x\in c\right\}|}$$

Accurate phase groups will have homogeneity values clustering around 0 and 1.

### Heterogeneity between phase groups

In addition to having a consistent haplotype source within phase groups, accurate phasing is reflected by proper segregation of haplotypes between complementary phase groups, *g_m _*and *g_n_*. To assess this metric, we compute the difference in the ratio of haplotype A reads between complementary phase groups as

$$het\left(g_{m},g_{n}\right)=\left|\frac{\sum_{c\in g_{m}}|\left\{x|x\in c\wedge x\in A\right\}|}{\sum_{c\in g_{m}}|\left\{x|x\in c\right\}|}-\frac{\sum_{c\in g_{n}}|\left\{x|x\in c\wedge x\in A\right\}|}{\sum_{c\in g_{n}}|\left\{x|x\in c\right\}|}\right|$$

Accurate phase groups result in heterogeneity values clustering around 1.

## Results

We validated our algorithm on a synthetic example in order to assess the accuracy of haplotype-specificity in assembly and scaffold phasing. We synthetically generated a 4.94-Mb diploid genome with a 0.04 heterozygosity rate. Haplotype A was the first 4.94 Mb of *Homo sapiens *chromosome 20 (gi 27501067). We derived haplotype B from this sequence using HapMaker [33]. We used ART v 1.3.1 [34] to generate synthetic error-free 75-bp paired reads from 400-bp inserts and from 1000-bp inserts at 40x coverage each (ART cannot simulate reads longer than 75 bp). We assembled reads using an overlap-layout consensus assembler Newbler 2.6 (-mi 100, -nohet). Only reads from 400-bp fragments were used for scaffolding. Bowtie 0.12.7 [35] was used for aligning reads to contigs (-v 3 -a -m 1 -f).

In all 21,358 contigs were combined into 1,252 scaffolds. There were 1,079 perfect bubbles (i.e., homolotig pairs) identified for training and another 3,651 contig pairs classified as homolotigs. Of these 4,187 homolotig pairs (88.5%) were phased into a total of 675 phase groups. In total, 3,275,028 non-N bases (91.0% percent of heterotig-classified bases) were phased.

**Figure 4:**
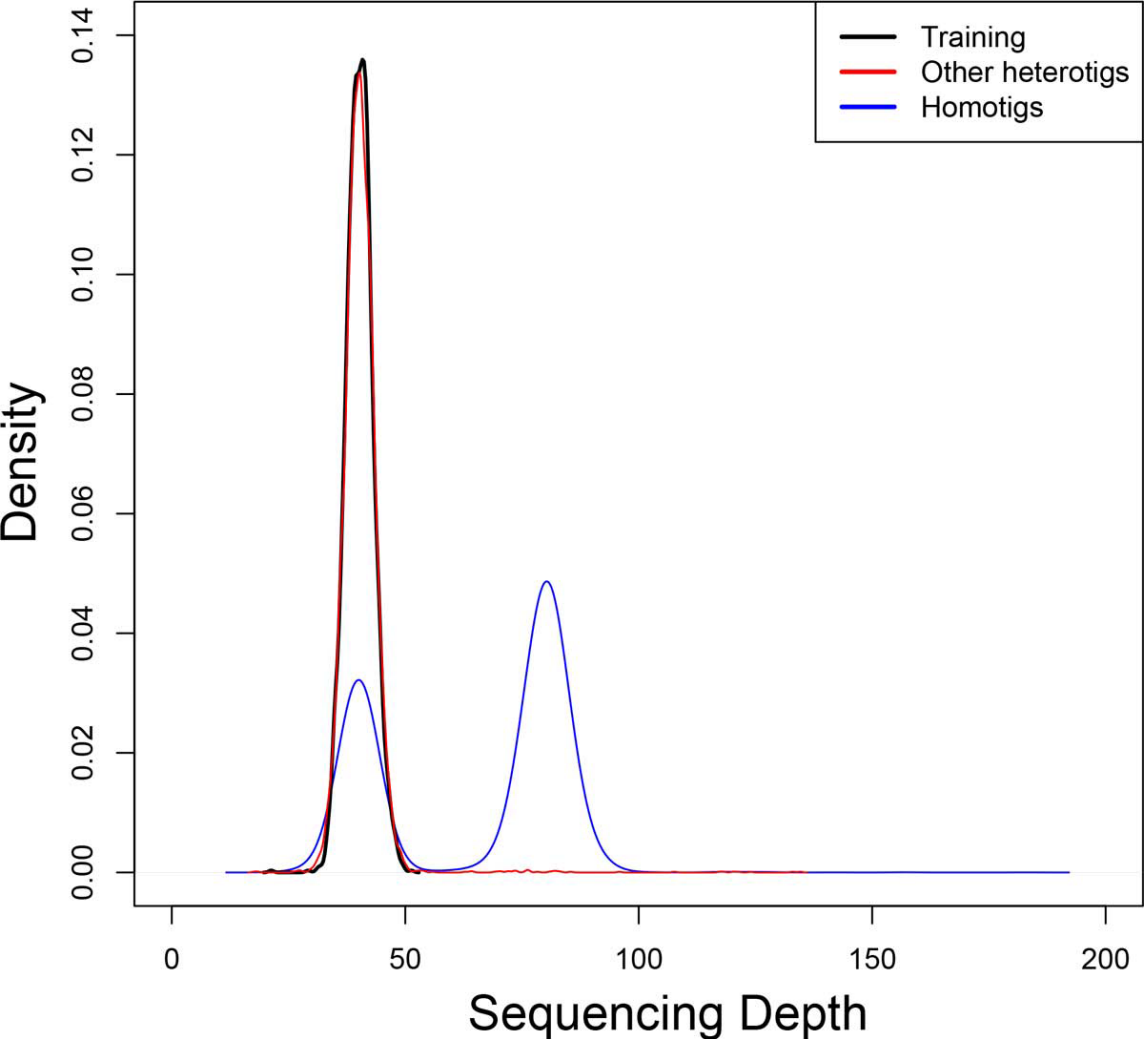
**Contig Sequencing Depth**. That reads from predicted heterotigs reflect a haploid sequencing depth indicates that haplotypes are segregating during assembly. The algorithm favors high precision (no false heterotigs) at some cost to recall (a few misclassified heterotigs), as evidenced by the blue peak at haploid sequencing depth.

**Figure 5:**
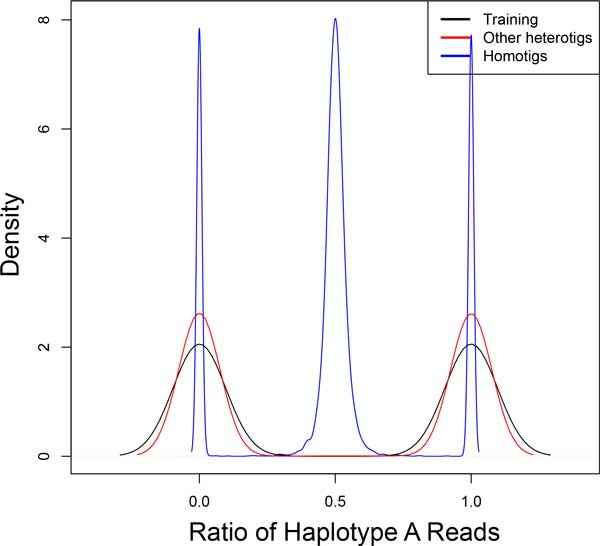
**Contig Homogeneity**. Reads from predicted heterotigs come either entirely from haplotype A or entirely from haplotype B, indicating that haplotypes are assembling correctly. We again see high precision with a few mislabeled homotigs (blue peaks at 0 and 1).

The results of diploid scaffolding using ScaffoldScaffolder are seen in Figures 4 through 7. Figures 4 and 5 demonstrate several results. First, looking at all contigs, regardless of their classification, we see two very clear categories: 1) homotigs, whose sequencing depth centers roughly at 80x coverage and whose haplotype A ratio centers at 0.5; and 2) heterotigs, whose sequencing depth centers at about 40x and whose haplotype A ratio centers at 0 and 1. This result suggests that a strict parameterization of the assembler successfully segregates heterozygous reads and combines homozygous reads. Second, we see that the contigs selected as "training" (which we assumed were heterotigs) fall entirely into the heterotig camp, suggesting that our method for selecting training examples is highly effective. Third, we see that we classify other heterotigs with high precision (99.7% of 7,302 classified heterotigs had sequencing depth *<*60), at some cost to recall (61.1% of 11,905 contigs with depth *<*60 were classified). Why we fail to classify all true heterotigs (i.e., those with depth *<*60) requires further investigation.

**Figure 6:**
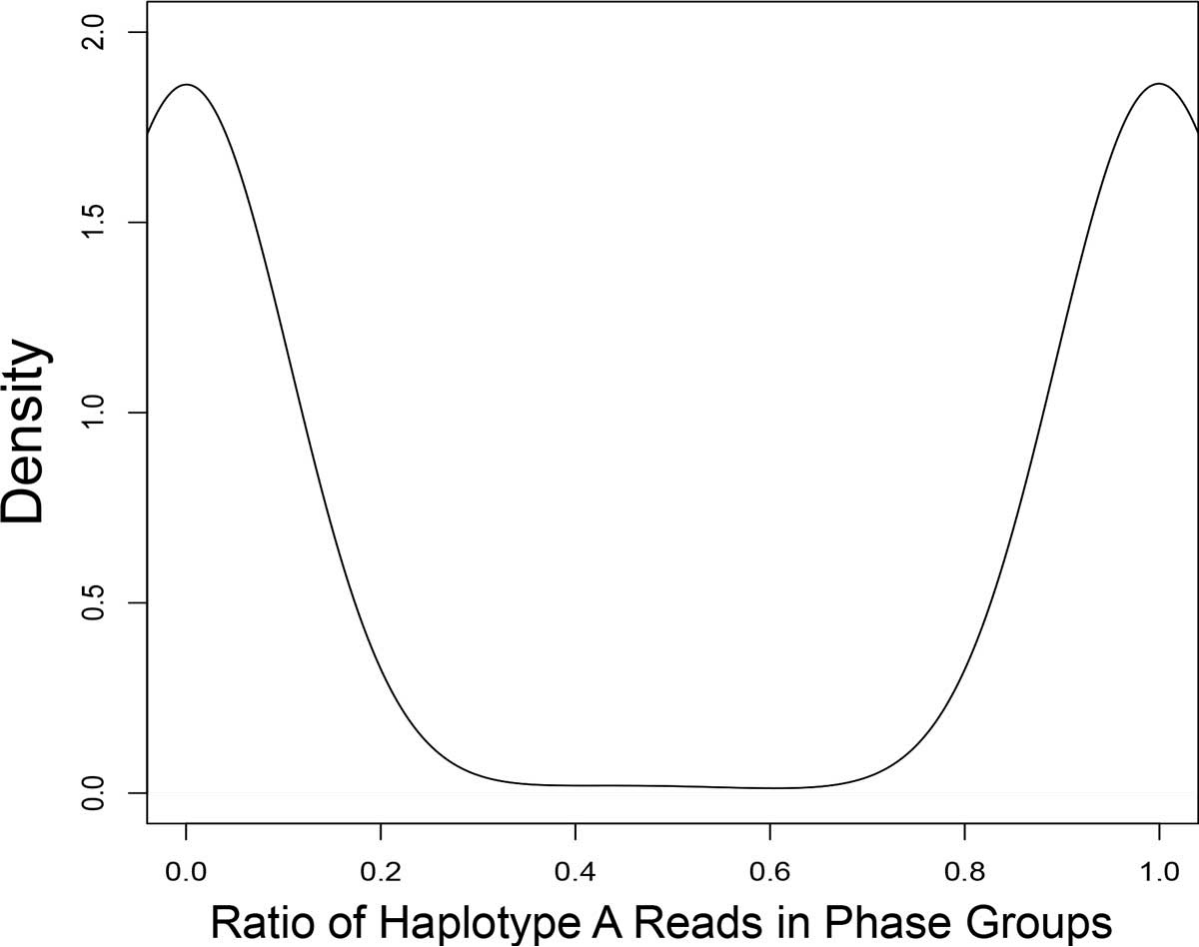
**Phase Group Homogeneity**. As with contig homogeneity, we observe that reads from commonly-phased contigs derive either entirely from haplotype A or entirely from haplotype B. This indicates accurate phasing.

**Figure 7:**
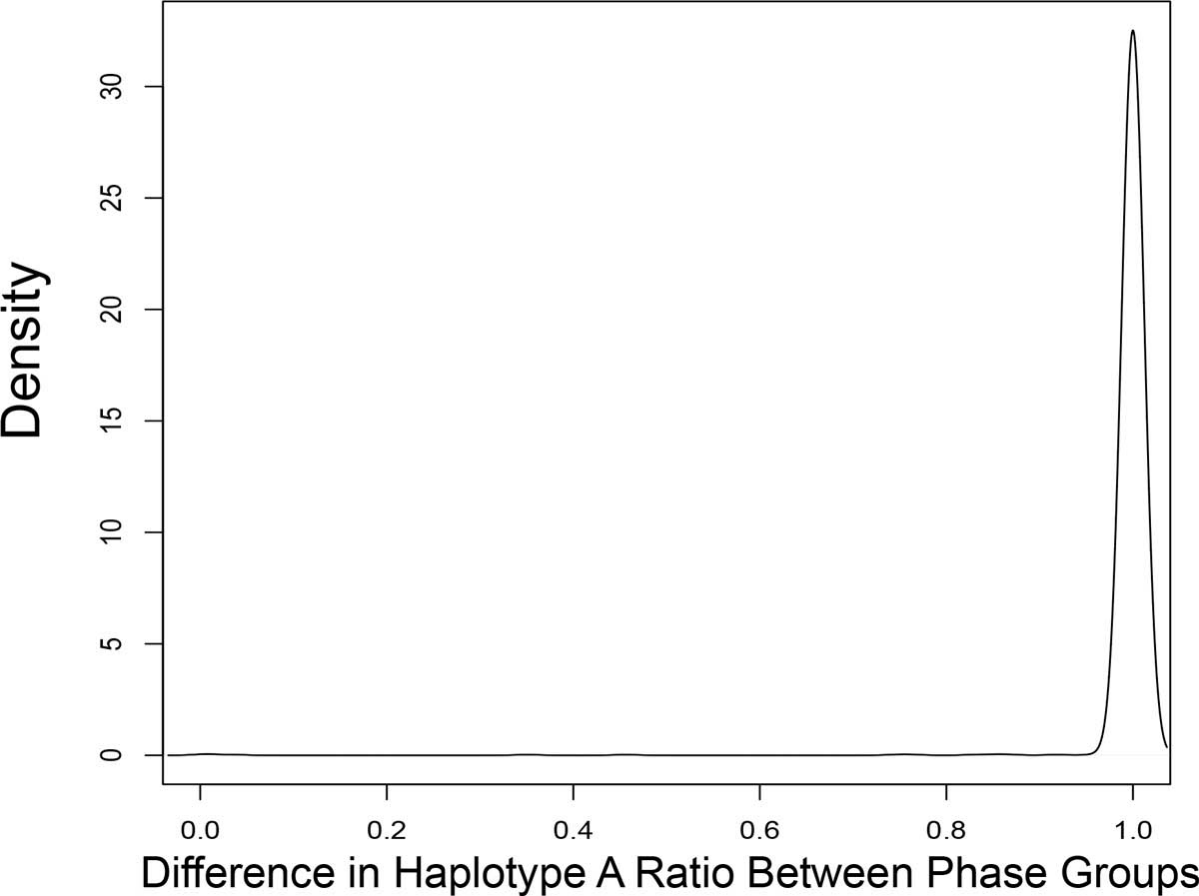
**Heterogeneity between Phase Groups**. Results indicate a complete and accurate segregation of haplotypes between complementary phase groups.

Figures 6 and 7 demonstrate the accuracy of phasing in ScaffoldScaffolder. Figure 6 demonstrates that *within *phase groups, reads derive either entirely from haplotype A or haplotype B. Figure 7 shows complete heterogeneity *between *homologous phase groups, meaning there are two haplotypes represented and cleanly segregated.

Overall these results indicate that at least given a highly conservative input dataset, the intuition and implementation of the algorithm are capable of effectively scaffolding and phasing diploid genomes. In the future we plan to perform more rigorous testing and comparative analysis on real datasets. It should also be noted that the algorithm as it currently stands is not designed not accommodate rearrangements or inversions.

## Conclusions

*De novo *diploid genome assembly is a burgeoning research area with exciting implications. We have presented ScaffoldScaffolder, a haplotype-aware scaffolding algorithm for diploid genomes. We have demonstrated the viability of using bubbles to identify heterozygous homologous contigs, which we term homolotigs. We have also shown that machine learning classification trained on these homolotig pairs can be used effectively for identifying homologous sequences elsewhere in the data with high precision (assuming error-free reads).

In addition to laying out the algorithm of ScaffoldScaffolder, we have defined four metrics which are indicative of diploid assembly quality when run on synthetic data: contig sequencing depth, contig homogeneity, phase group homogeneity, and heterogeneity between phase groups. More work is required to comparatively analyze this approach on real data with various parameters and classifiers against other diploid genome assembly methods (of which there are currently very few). However, the initial results of ScaffoldScaffolder supply validity to the idea of employing machine learning in the difficult task of diploid genome assembly.

## Competing interests

The authors declare that they have no competing interests.

## Authors' contributions

PMB conceived of the study, carried out experiments, and drafted the manuscript. MSF, JCP, MJC, and, QS contributed substantially to conception and design. NO contributed to data acquisition, synthetic data preparation, and manuscript editing. CO aided substantially in analysis, interpretation, and presentation of results.

## Declarations

Funding used to cover the publishing charge also comes from NIH grant R01 HG005692.

This article has been published as part of *BMC Bioinformatics *Volume 16 Supplement 7, 2015: Selected articles from The 11th Annual Biotechnology and Bioinformatics Symposium (BIOT-2014): Bioinformatics. The full contents of the supplement are available online at http://www.biomedcentral.com/bmcbioinformatics/supplements/16/S7.
